# Prognostic implications of tricuspid annular plane systolic excursion/pulmonary arterial systolic pressure ratio in septic shock patients

**DOI:** 10.1186/s12947-020-00198-y

**Published:** 2020-06-12

**Authors:** Hongmin Zhang, Hui Lian, Qing Zhang, Xiukai Chen, Xiaoting Wang, Dawei Liu

**Affiliations:** 1 Department of Critical Care Medicine, Peking Union Medical College Hospital, Chinese Academy of Medical Sciences, 1# Shuai Fu Yuan, Dong Cheng District, Beijing, 100730 China; 2 Department of Health Care, Peking Union Medical College Hospital, Chinese Academy of Medical Sciences and Peking Union Medical College, Beijing, China; 3 Department of Critical Care Medicine, Peking Union Medical College Hospital, Chinese Academy of Medical Sciences and Peking Union Medical College, Beijing, China; 4 Pittsburgh Heart, Lung, Blood and Vascular Institute, University of Pittsburgh, School of Medicine, Pittsburgh, PA USA

**Keywords:** Echocardiography, Tricuspid annular plane systolic excursion, Pulmonary arterial pressure, Septic shock, Prognosis

## Abstract

**Background:**

To explore the association between the ratio of tricuspid annular plane systolic excursion (TAPSE) and pulmonary arterial systolic pressure (PASP), and long- and short-term outcomes in mechanically ventilated septic shock patients.

**Methods:**

Septic shock patients admitted to the intensive care unit (ICU) were screened for enrollment. Echocardiographic parameters including TAPSE and tricuspid regurgitation velocity, haemodynamic and respiratory parameters, and prognostic data were obtained.

**Results:**

One hundred eighteen subjects were enrolled in this study, among whom 75 survived and 43 died at the one-year follow-up. ROC curve analysis revealed that the TAPSE/PASP ratio was able to assess one-year all-cause mortality with an area under the curve of 0.817 (95% CI: 0.739–0.896, *p* < 0.001) and the optimal cutoff value was 0.50 mm/mmHg. Kaplan-Meier survival analysis showed that one-year all-cause mortality was significantly higher in patients with TAPSE/PASP ≤0.5 mm/mmHg than in patients with TAPSE/PASP > 0.5 mm/mmHg (log-rank 32.934, *p* < 0.001). According to the Cox regression survival analyses, the TAPSE/PASP ratio was independently associated with one-year all-cause mortality (HR 0.007, 95% CI:0.000–0.162, *p* = 0.002) and ICU mortality (HR 0.027, 95% CI:0.001–0.530, *p* = 0.017). According to the multivariable analysis, the TAPSE/PASP ratio was an independent variable associated with mechanical ventilation (MV) duration (standard coefficient − 0.240, *p* = 0.010).

**Conclusion:**

The TAPSE/PASP ratio demonstrated prognostic value for one-year all-cause mortality, ICU mortality and MV duration in mechanically ventilated septic shock patients.

## Introduction

Septic shock is a major health problem, affecting millions of people around the world each year and having high morbidity and mortality [1]. Septic cardiomyopathy, which is usually diagnosed via echocardiography, has a high incidence in septic shock patients. Although the definition of septic cardiomyopathy is based on the left ventricular ejection fraction (LVEF), both ventricles can be affected [2, 3]. Furthermore, it has been increasingly recognized that right ventricular (RV) systolic dysfunction is associated with long-term prognosis in septic patients [4, 5].

The right ventricle (RV) is anatomically and functionally different from the left ventricle and is more prone to be affected by the alterations in afterload [6]. In addition to the decrease in intrinsic RV contractile function in septic shock, an increase in RV afterload is also common, resulting from complications including acute respiratory distress syndrome (ARDS), concomitant LV dysfunction, or positive pressure ventilation [7–9]. Winkelhorst and his colleagues, in a recent study, indicated that RVEF was associated with one-year mortality in septic patients. Their data also showed that the pulmonary arterial pressure was significantly higher in patients with low RVEF than in patients with high RVEF [5]. Thus, a combined assessment of RV systolic function and its afterload by right ventricular-pulmonary arterial coupling would potentially provide additional physiological information in septic patients.

The ratio of tricuspid annular plane systolic excursion (TAPSE) and pulmonary arterial systolic pressure (PASP) is deemed as an indicator of right ventricular-pulmonary arterial coupling [10]. TAPSE is a simple and reproducible parameter of RV systolic function with low interobserver variability, even in patients with raised right-sided pressures [11, 12]. PASP can be reliably determined from the peak tricuspid regurgitation velocity in the majority of patients [13]. The TAPSE/PASP ratio was found to be associated with mortality in patients with pulmonary arterial hypertension or heart failure [10, 14–16]. However, whether TAPSE/PASP ratio is related to the prognosis of septic shock patients has not been investigated. We hypothesize that right ventricular-pulmonary arterial coupling can also be compromised in septic shock and that the TAPSE/PASP ratio is of prognostic value among these patients. Accordingly, the aim of the present study was to explore the association between the TAPSE/PASP ratio and the long- and short-term prognoses of mechanically ventilated septic shock patients.

## Patients and methods

### Study population

This prospective observational study was conducted at a tertiary hospital intensive care unit (ICU). Patients admitted from 1 May 2017 to 1 October 2018 were screened for enrolment within the first 24 h after admission.

We enrolled septic shock patients who were on mechanical ventilation. Septic shock was defined as sepsis with persisting hypotension requiring vasopressors to maintain a mean arterial pressure equal to or above 65 mmHg and having a serum lactate level above 2 mmol/L despite adequate volume resuscitation [17].

Patients were excluded if they had any of the following criteria: age less than 18 years; acute coronary syndrome within 1 week; rhythm characteristics of atrial fibrillation; prosthetic valves or valvular diseases such as severe mitral, aortic or tricuspid stenosis or regurgitation; moderate to severe chronic pulmonary hypertension; an inadequate echocardiographic images for measurement; no monitoring of central venous pressure (CVP); without informed consent; withholding of life support; and loss of follow up.

The study was conducted in compliance with the Declaration of Helsinki and was approved by the ethics committee of our institution (Approval No. ZS-1422). Informed consent was obtained from the next of kin.

### Echocardiography

Echocardiograms were recorded within the first 24 h of ICU admission using an echocardiograph (X-Porte, SonoSite, USA) with a 2.5-MHz phased-array probe. Images were saved for offline analysis. Two physicians who were experienced in echocardiography performed the echo examination. Electrocardiograms were recorded continuously during the examination. Three cardiac cycles were analysed and averaged. M-mode and Doppler echocardiographic measurements were taken according to standard protocols.

TAPSE was obtained in the apical 4-chamber view by positioning the M-mode cursor along the lateral part of the tricuspid valve ring and measuring the difference between the highest and lowest points of the M-mode sinusoid wave [18]. The right ventricular outflow tract (RVOT) dimensions were obtained from the parasternal short-axis view at the level of the aortic root. The velocity of tricuspid regurgitation (TR) was measured in the RV inflow view, apical 4-chamber view and aortic short-axis view via continuous wave Doppler, and the highest value was chosen. The pulmonary arterial systolic pressure (PASP) was calculated by the following equation: PASP = 4 × (TR velocity)^2^ + CVP [13]. RVOT fractional shortening (RVOT-FS) was calculated as (dimension at end-diastole – end-systole)/end-diastole [19]. The left ventricular ejection fraction (LVEF) was obtained using a modified biplane Simpson’s method from the apical two- and four-chamber views. The mitral annular plane systolic excursion (MAPSE) was obtained from the apical 4-chamber view by positioning the cursor along the lateral mitral ring. The mitral e’ velocity was measured with tissue Doppler imaging by placing the sample volume on the lateral and medial mitral annulus, and the averaged value from both annuli was chosen.

### Other parameters collected

Demographic information and the diagnosis, Acute Physiology and Chronic Health Evaluation (APACHE) II score, Sequential Organ Failure Assessment (SOFA) score, comorbidities, maximum norepinephrine (NE) dose, arterial blood lactate level at ICU admission and the timing of the echo examination were collected for all patients. Each patient’s heart rate (HR), mean arterial pressure (MAP), CVP, total fluid infusion, positive end-expiratory pressure (PEEP), and plateau pressure (Pplat) were also collected at the time of the echo examination.

### Endpoints

The primary outcome was one-year all-cause mortality. The secondary outcomes included ICU mortality, ICU length of stay, and mechanical ventilation (MV) duration. One-year mortality was obtained via a telephone survey of the relatives. The other data were obtained from the medical records.

### Statistical analyses

The statistical analysis was performed using the statistical software package SPSS 13.0 (SPSS, Inc., Chicago, Illinois, USA). Continuous variables are expressed as the mean ± SD or as the median and the interquartile range. Categorical variables are presented as frequencies and percentages. The distributions of the continuous values were assessed for normality by the Kolmogorov-Smirnov test. Differences among groups were assessed by Student’s unpaired *t* test, the Mann-Whitney U test, the Kruskal-Wallis test, the chi-squared test, or Fisher’s exact test, as appropriate. Spearman’s correlation coefficients and their corresponding *p* values were calculated to assess the variable relationships. Receiver operating characteristic (ROC) curve analysis was used to identify the optimal cutoff value of the TAPSE/PASP ratio in the assessment of one-year all-cause mortality. Cumulative survival curves of the one-year follow-up were estimated with the Kaplan-Meier method, and the effect of the TAPSE/PASP ratio on the survival probability was compared between groups using a log-rank test. Prognostic factors for one-year all-cause mortality and ICU mortality were determined using the Cox regression model. The following variables were considered for the survival analysis: age, APACHEII, SOFA, NE dose, Pplat, TAPSE, PASP, TAPSE/ PASP ratio, RVOT-FS, LVEF, MAPSE, and e’. The variables that had *p* < 0.25 in the univariable model were included in the multivariable model and the hazard ratio was calculated, together with its 95% confidence intervals. Given the collinearity between TAPSE, PASP and the TAPSE/PASP ratio, separate Cox regression models were performed. Multivariable linear regression analysis was performed to assess the independent associations of the general characteristics and the echocardiographic parameters with MV duration and ICU length of stay. As MV duration and ICU length of stay did not fit a Gaussian distribution, a logarithm was taken. Variables were assessed for collinearity prior to inclusion in the model. Intra-observer and interobserver variabilities in TAPSE, RVOT-FS, LVEF, MAPSE, and e’ were assessed in 20 randomly selected patients and were tested using both paired t tests and intraclass correlation coefficients (ICCs). An ICC > 0.8 was considered excellent agreement. Two-tailed *p* < 0.05 was considered significant.

## Results

### Measurement variability

The intra-observer variabilities in TAPSE, RVOT-FS, LVEF, MAPSE and mitral e’ velocity were minimal. The interobserver variability analysis revealed that the ICCs for TAPSE, TR velocity, RVOT-FS, LVEF, MAPSE, and e’ were 0.966 (95% CI: 0.915–0.987),0.926 (95% CI: 0.743–0.974) 0.910 (95% CI: 0.744–0.970), 0.900 (95% CI: 0.759–0.960), 0.959 (95% CI: 0.891–0.985), and 0.924 (95% CI: 0.863–0.979), respectively.

### General characteristics of all patients

**Fig. 1:**
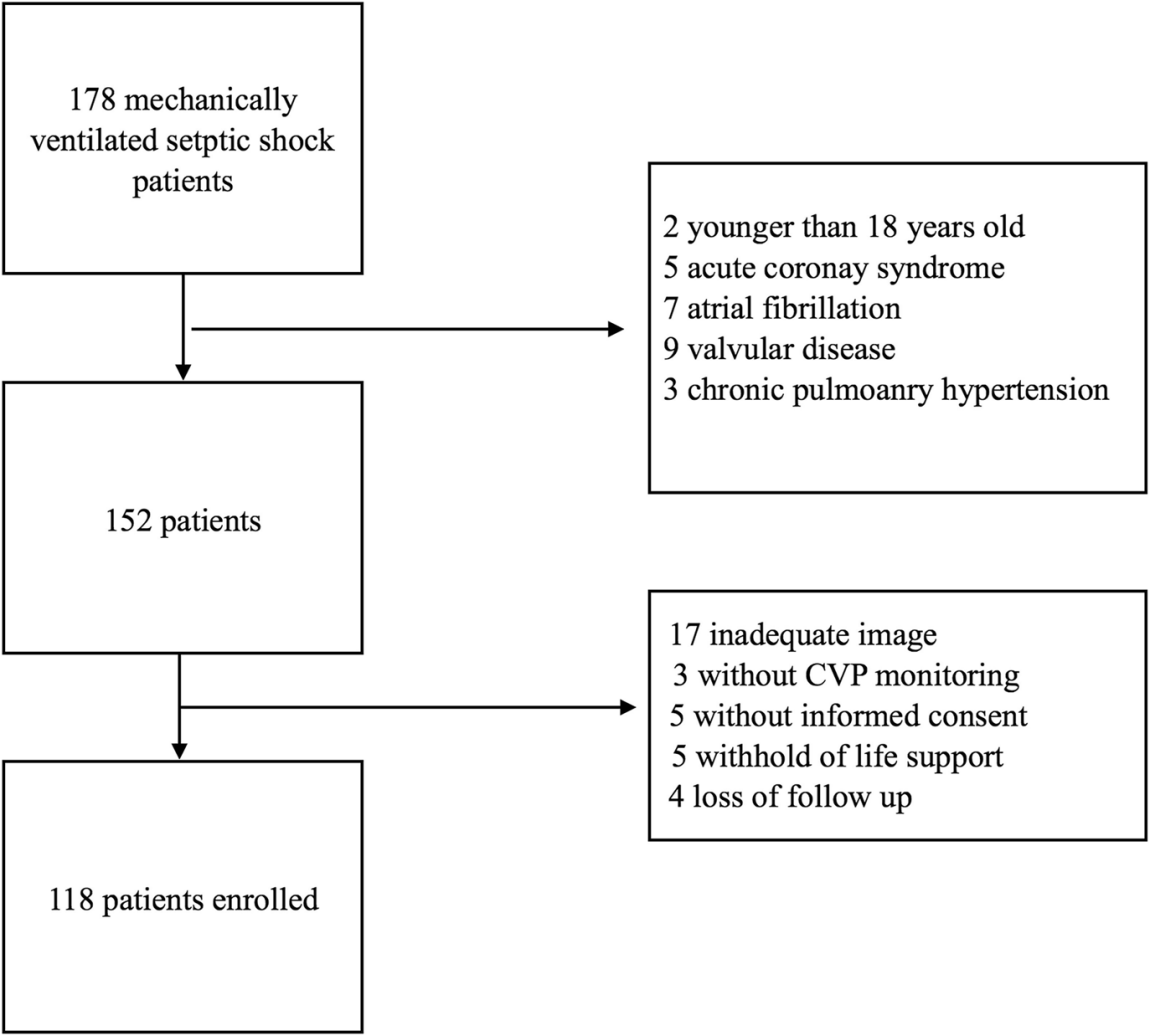
Flow chart of the study

A total of 178 patients were screened for enrolment. Sixty patients were excluded because of diagnoses that could be confounding factors, inadequate images, a lack of informed consent, or withholding of life support or because they were lost to follow-up (Fig. 1). One hundred eighteen patients were enrolled in this study, among whom 75 survived and 43 died at the one-year follow-up. The general characteristics are listed in Table 1. The survivors and non-survivors had similar ages, sex proportions, diagnoses and comorbidities. Compared to survivors, non-survivors had higher APACHE II scores (*p* < 0.001) and SOFA scores (*p* < 0.001) as well as higher maximum NE doses (*p* = 0.001).

**Table 1:** General characteristics

Categories	Survivors (*n* = 75)	Non-survivors (*n* = 43)	*p* value
Age (yr)	59.3 ± 15.3	67.6 ± 14.1	0.004
Sex (male, %)	47 (62.6%)	29 (67.4%)	0.590
APACHE II	18 (14, 22)	26 (19, 30)	< 0.001
SOFA	12 (10, 13)	14 (12, 16)	< 0.001
Diagnosis (n, %)			
Pneumonia	15 (20.0%)	13 (30.2%)	0.208
Abdominal infection	38 (50.7%)	17 (39.5%)	0.250
Biliary tract infection	4 (5.3%)	2 (4.7%)	0.863
CRBSI	4 (5.3%)	2 (4.7%)	0.863
Cellulitis	10 (13.3%)	5 (11.6%)	0.802
Others*	4 (5.3%)	4 (9.3%)	0.405
Comorbidities			
HTN	36 (48.0%)	25 (58.1%)	0.284
DM	28 (37.3%)	22 (51.2%)	0.143
CAD	14 (18.7%)	13 (30.2%)	0.147
CKD	5 (6.7%)	6 (14.0%)	0.194
COPD	7 (9.3%)	8 (18.6%)	0.151
Timing of echo (hr from admission)	12 (8,18)	13 (9, 16)	0.905
Maximum NE dose (μg/kg/min)	0.36 (0.18, 0.75)	0.81 (0.40, 1.61)	0.001
Fluid administered (ml)	2560 (1366, 3140)	2880 (1629, 3530)	0.766
Lactate (mmol/L)	3.5 (2.7, 4.2)	4.0 (3.3, 4.9)	0.185
MV duration (hr)	108 (67, 240)	203 (105, 340)	0.051
ICU length of stay (day)	7 (4, 12)	10 (6, 15)	0.233

*TAPSE* tricuspid annular plane systolic excursion, *APACHE* acute physiology and chronic health evaluation, *SOFA* sequential organ failure assessment, *UTI* urinary tract infection, *CRBSI* catheter related bloodstream infection, *HTN* hypertension, *DM* diabetes mellitus, *CAD* coronary arterial disease, *CKD* chronic kidney dysfunction, *COPD* chronic obstructive pulmonary disease, *NE* norepinephrine, *ICU* intensive care unit

^*^Others including urinary tract infection, intracranial, mediastinum infections

### Comparison of haemodynamic, respiratory and echocardiographic parameters between the survivors and non-survivors

The survivors and non-survivors had similar HR, MAP and PEEP levels. The Pplat level was higher in non-survivors than in survivors (*p* = 0.031). The CVP level was higher in non-survivors than in survivors, but the difference was not statistically significant (*p* = 0.074). Compared to survivors, non-survivors had lower TAPSE (*p* < 0.001), higher PASP (*p* = 0.003) and a lower TAPSE/PASP ratio (*p* < 0.001); non-survivors also had lower RVOT-FS (*p* = 0.042), MAPSE (*p* = 0.012) and mitral e’ velocity values (*p* = 0.043) (Table 2).

**Table 2:** Haemodynamic, respiratory and echocardiographic parameters of the two groups

	Survivors (n = 75)	Non-survivors (n = 43)	*p* Value
HR (bpm)	89 (80, 101)	107 (78, 116)	0.174
MAP (mmHg)	82 ± 4	80 ± 9	0.407
CVP (mmHg)	9 (8, 12)	10 (9, 13)	0.074
PEEP (cmH_2_O)	5 (5, 8)	6 (5, 8)	0.440
Pplat (cmH_2_O)	18 (16, 20)	19 (17, 22)	0.031
TAPSE (mm)	19.5 ± 5.1	15.1 ± 4.8	< 0.001
PASP (mmHg)	32.0 ± 9.6	37.9 ± 11.0	0.003
TAPSE/PASP (mm/mmHg)	0.61 (0.50, 0.81)	0.39 (0.31, 0.53)	< 0.001
RVOT-FS (%)	43 (34, 55)	38 (30, 48)	0.042
LVEF (%)	58 ± 13	57 ± 15	0.244
MAPSE (mm)	14.6 ± 4.6	9.9 ± 0.8	0.012
e’ (cm/s)	8.3 (6.9–10.6)	7.5 (4.6, 9.1)	0.043

*HR* heart rate, *MAP* mean arterial pressure, *CVP* central venous pressure, *PEEP* positive end-expiratory pressure, *Pplat* plateau pressure, *TAPSE* tricuspid annular plane systolic excursion, *PASP* pulmonary arterial systolic pressure, *RVOT-FS* right ventricular outflow tract fractional shortening, *LVEF* left ventricular ejection fraction, *MAPSE* mitral annular plane systolic excursion; e’: mitral e’ velocity

### Correlation between TAPSE and PASP and the relationship between the TAPSE/PASP ratio and TAPSE, RVOT-FS, LVEF, and MAPSE

**Fig. 2:**
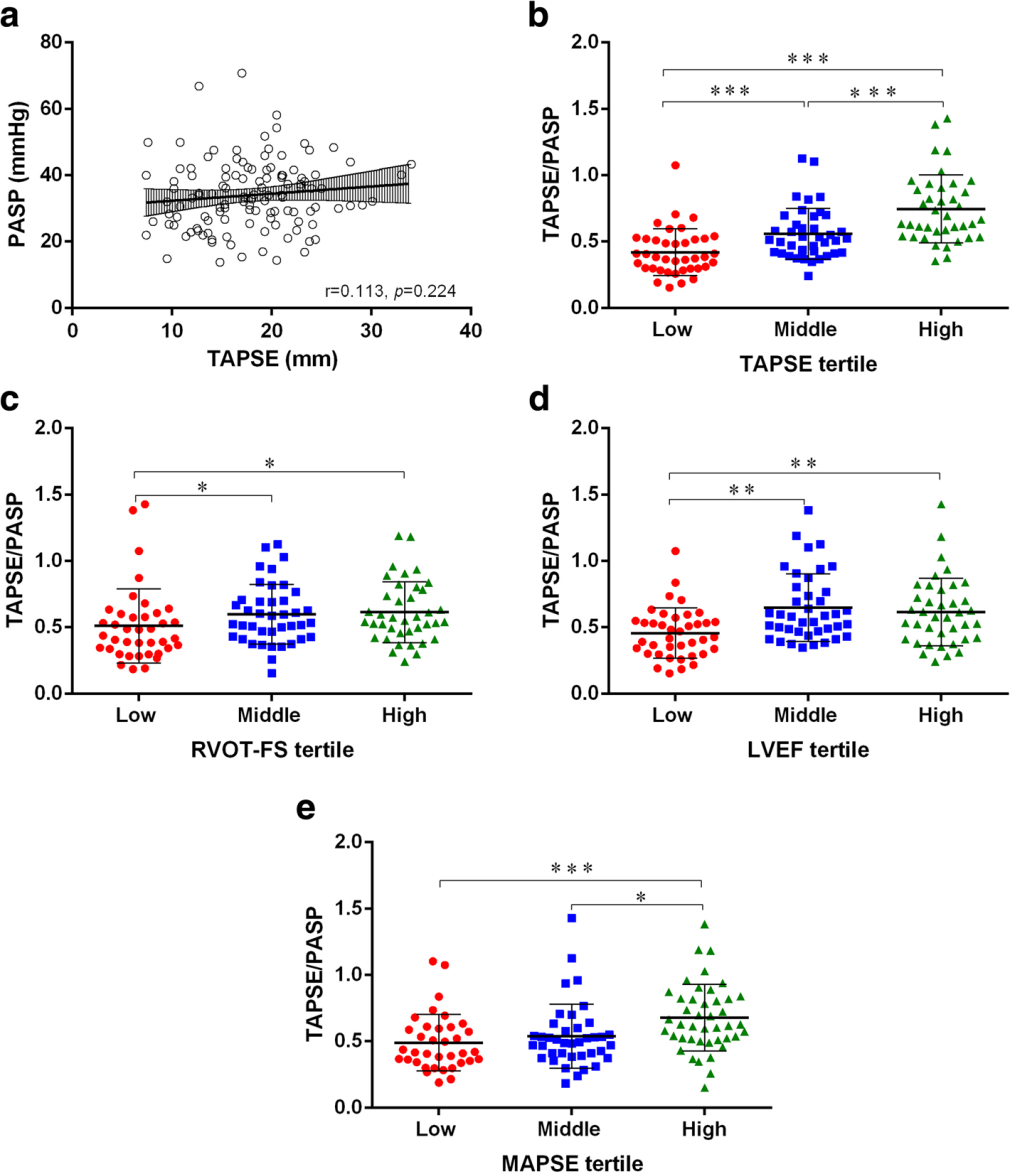
Correlation between TAPSE and PASP, relationships of TAPSE/PASP ratio with TAPSE, RVOT-FS, LVEF, and MAPSE. **a**. Correlation between the TAPSE and PASP. TAPSE was not associated with PASP, r = 0.113, *p* = 0.224. **b**. Relationship of TAPSE/PASP ratio with TAPSE tertiles (low: TAPSE ≤15.0 mm; middle: TAPSE 15.1 mm–19.9 mm; high: TAPSE ≥20.0 mm). **c**. Relationship of TAPSE/PASP ratio with RVOT-FS tertiles (low: RVOT-FS ≤ 34%; middle: RVOT-FS 35–47%; high: RVOT-FS ≥ 48%). **d**. Relationship of TAPSE/PASP ratio with LVEF tertiles (low: LVEF ≤50%; middle: LVEF 51–62%; high: LVEF ≥63%). e. Relationship of TAPSE/PASP ratio with MAPSE tertiles (low: MAPSE ≤11.0 mm; middle: MAPSE 11.1 mm–14.0 mm; high: MAPSE ≥14.1 mm). Lines in **b**-**e** indicate median and interquartile range, * *p* < 0.05, ** *p* < 0.01, *** *p* < 0.001(Kruskal-Wallis test). TAPSE: tricuspid annular plane systolic excursion; PASP: pulmonary arterial systolic pressure; RVOT-FS: right ventricular outflow tract fractional shortening; LVEF: left ventricular ejection fraction; MAPSE: mitral annular plane systolic excursion

TAPSE was not associated with PASP (r = 0.113, *p* = 0.224) (Fig. 2a). We divided patients into three tertiles according to TAPSE, RVOT-FS, LVEF and MAPSE. The TAPSE/PASP ratio was able to differentiate between tertiles of TAPSE (Fig. 2b). Patients with low RVOT-FS had a lower TAPSE/PASP ratio than those with middle and high RVOT-FS (Fig. 2c). Patients with low LVEF had a lower TAPSE/PASP ratio than those with middle and high LVEF (Fig. 2d). Compared with the high tertile, the low MAPSE tertile demonstrated a significantly lower TAPSE/PASP ratio (Fig. 2e).

### Primary outcome

**Fig. 3:**
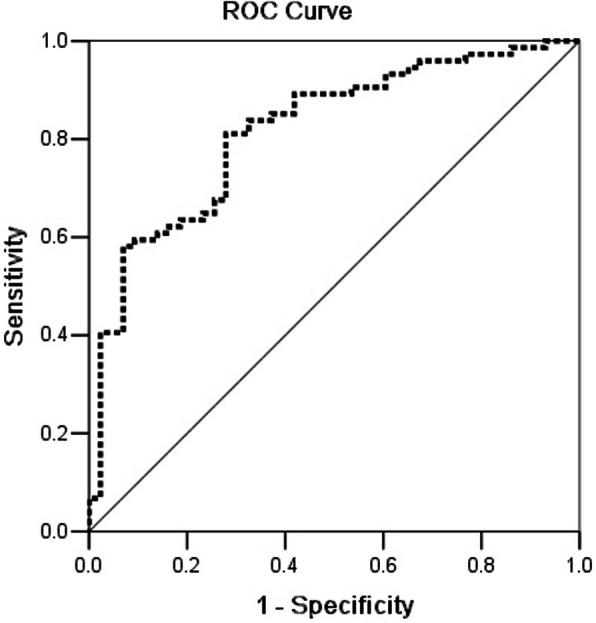
ROC curve analysis of TAPSE/PASP ratio for one-year all-cause mortality. The area under the curve was 0.817 (95% CI: 0.739–0.896, *p* < 0.001). TAPSE: tricuspid annular plane systolic excursion; PASP: pulmonary arterial systolic pressure

To determine the cutoff value of the TAPSE/PASP ratio in the assessment of one-year all-cause mortality, ROC curves were generated (Fig. 3). The area under the curve (AUC) for the TAPSE/PASP ratio in order to assess one-year all-cause mortality was 0.817 (95% CI: 0.739–0.896, *p* < 0.001) and the optimal cutoff value was 0.50 mm/mmHg (Table 3).

**Table 3:** ROC analysis of TAPSE/PASP ratio in the prediction of one-year all-cause mortality

	AUC	95%CI	*p*	Optimal cutoff	Sensitivity	Specificity	PPV	NPV
TAPSE/PASP (mm/mmHg)	0.817	0.739–0.896	< 0.001	0.50	74.3%	72.1%	60.4%	83.1%

*TAPSE* tricuspid annular plane systolic excursion, *PASP* pulmonary arterial systolic pressure, *AUC* area under curve, *PPV* positive predictive value, *NPV* negative predictive value

**Fig. 4:**
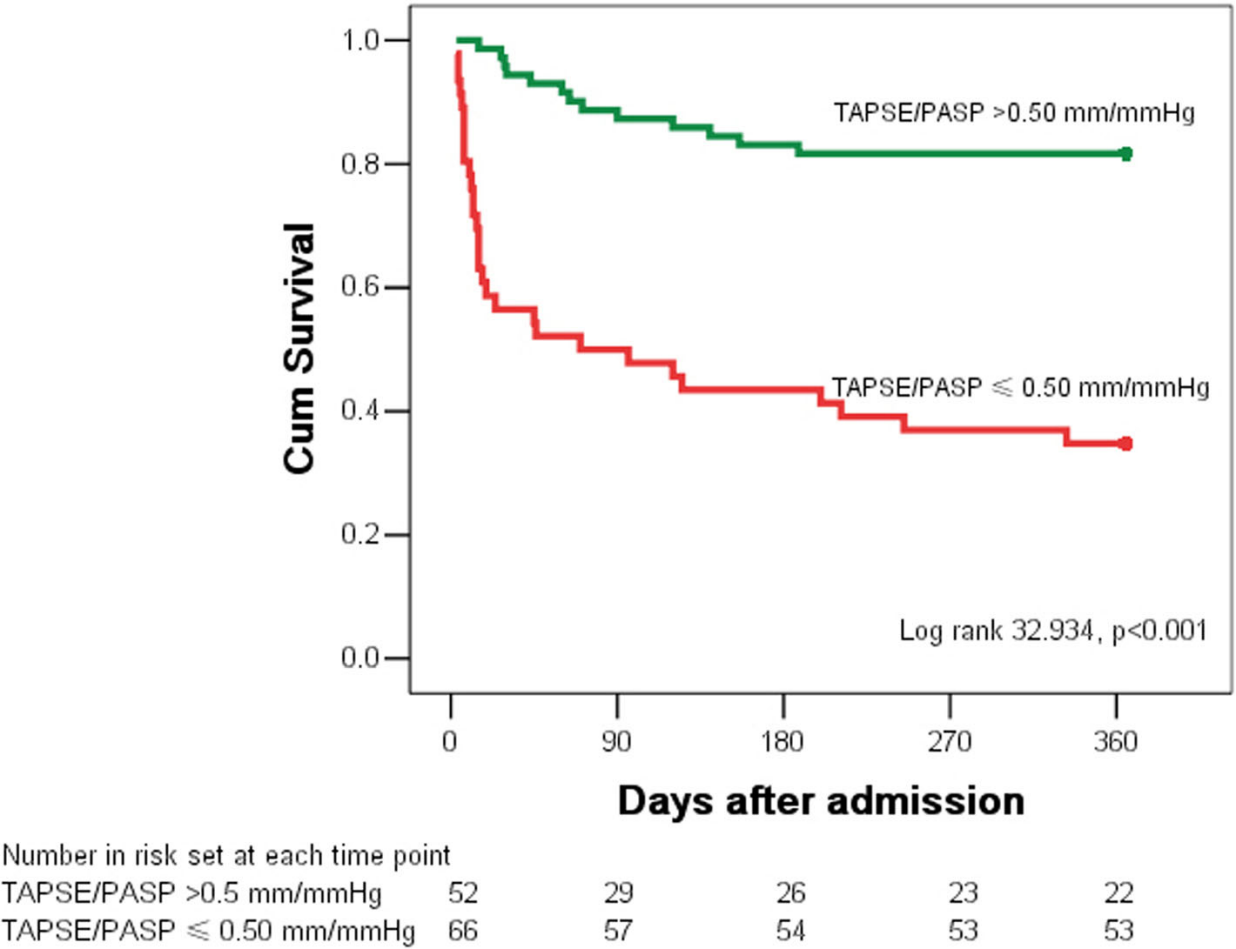
The Kaplan-Meier curves for estimated survival showed that one-year all-cause mortality was significantly higher in patients with TAPSE/PASP ≤0.50 mm/mmHg than in patients with TAPSE/PASP > 0.50 mm/mmHg (log-rank: 32.934, *p* < 0.001). TAPSE: tricuspid annular plane systolic excursion; PASP: pulmonary arterial systolic pressure

At the one-year follow-up, 19.7% (13/66) of patients with TAPSE/PASP > 0.50 mm/mmHg died; whereas 57.7% (30/52) of patients with TAPSE/PASP ≤0.50 mm/mmHg died. The Kaplan-Meier curves for estimated survival showed that one-year all-cause mortality was significantly higher in patients with TAPSE/PASP ≤0.50 mm/mmHg than in patients with TAPSE/PASP > 0.50 mm/mmHg (log-rank: 32.934, *p* < 0.001) (Fig. 4).

According to the Cox regression survival analysis, after adjusting for age, APACHE II, SOFA, NE dose, Pplat, RVOT-FS, MAPSE, LVEF and e’, the TAPSE/PASP ratio was independently associated with one-year all-cause mortality (HR 0.007, 95% CI:0.000–0.162, *p* = 0.002) (Table 4).

**Table 4:** Factors associated with one-year all-cause mortality

	Hazard Ratio	95%CI	*p* Value
Univariable analysis			
Age	1.030	1.008–1.053	0.009
APACHEII	1.087	1.050–1.125	< 0.001
SOFA	1.202	1.089–1.327	< 0.001
NE dose	1.897	1.348–2.671	0.001
Pplat	1.101	1.018–1.192	0.017
CVP	1.083	0.992–1.182	0.074
TAPSE	0.269	0.144–0.502	< 0.001
PASP	1.047	1.018–1.077	0.001
TAPSE/PASP	0.006	0.001–0.044	< 0.001
RVOT-FS	0.980	0.956–1.004	0.096
MAPSE	0.350	0.141–0.870	0.024
LVEF	0.987	0.965–1.009	0.239
e’	0.882	0.792–0.982	0.022
Multivariable analysis 1			
LVEF	1.019	0.985–1.055	0.272
APACHEII	1.064	1.008–1.123	0.025
PASP	1.046	1.012–1.081	0.008
TAPSE	0.248	0.083–0.741	0.013
Multivariable analysis 2			
LVEF	1.018	0.986–1.051	0.271
APACHEII	1.058	1.006–1.113	0.029
SOFA	1.168	1.027–1.327	0.018
TAPSE/PASP	0.007	0.000–0.162	0.002

*APACHE* acute physiology and chronic health evaluation, *SOFA* sequential organ failure assessment, *NE* norepinephrine, *Pplat* plateau pressure, *CVP* central venous pressure, *TAPSE* tricuspid annular plane systolic excursion, *PASP* pulmonary arterial systolic pressure, *RVOT-FS* right ventricular outflow tract fractional shortening, *LVEF* left ventricular ejection fraction, *MAPSE* mitral annular plane systolic excursion; e’: mitral e’ velocity

### Secondary outcomes

The ICU mortality rates in patients with TAPSE/PASP > 0.50 mm/mmHg and patients with TAPSE/PASP ≤0.50 mm/mmHg were 6.1% (4/66) and 40.4% (21/52), respectively. The Cox regression analysis showed that the TAPSE/PASP ratio was independently associated with ICU mortality (HR 0.027, 95% CI:0.001–0.530, *p* = 0.017) (Supplemental Table 1). According to the multivariable analysis, the TAPSE/PASP ratio was not an independent variable associated with ICU length of stay but was independently associated with MV duration (standard coefficient − 0.240, *p* = 0.010) (Supplemental Tables 2, 3).

## Discussion

In this study, we assessed the TAPSE/PASP ratio in mechanically ventilated septic shock patients and investigated its association with the prognosis of these patients. We observed that the TAPSE/PASP ratio was an independent predictor of one-year all-cause mortality. We also noticed that the TAPSE/PASP ratio was associated with ICU mortality and MV duration in this cohort of patients.

Our study exclusively enrolled mechanically ventilated septic shock patients. This is different from prior studies, which also incorporated patients who were not in a shock state and those who were not on mechanical ventilation [4, 5, 20–22]. Although the ICU houses fewer than 10% of total hospital beds, ICU care accounts for one-third of total health care costs [23]. Furthermore, ICU survivors, particularly those with longer MV durations and ICU stays, often suffer from ICU-acquired weakness and physical dysfunction [24, 25]. Thus, studies regarding the MV duration, ICU length of stay and long-term survival of mechanically ventilated septic shock patients are necessary to improve the short-term and long-term prognoses and reduce the costs of these patients. General assessment scores of illness severity, such as SOFA and APACHE scores, may not be sensitive enough to reflect the prognosis of patients with different heart functions [4, 26]. Our results showed that, if APACHE II and SOFA scores as well as other echocardiographic variables were included in the same model, the TAPSE/PASP ratio was still of prognostic value in these patients.

The TAPSE/PASP ratio reflects the interaction between RV systolic function and its afterload. As a feasible and reproducible RV function parameter, TAPSE correlated well with RV ejection fraction [11]. TAPSE appears to be reproducible and has been proven to be a strong predictor of prognosis in heart failure and critically ill patients [26–28]. Interestingly, PASP was also found to be related to the prognosis of heart failure patients [9, 29]. In comparison with LV, RV was more sensitive to the afterload alteration [30]. Several factors might have contributed to the increase in PASP in septic shock patients. Apparently, positive pressure ventilation increases pulmonary vascular resistance [8]. Furthermore, ARDS is a common complication of severe sepsis and septic shock [7]. Even with lung protection ventilation, ARDS can challenge the RV with an incidence in acute cor pulmonale (ACP) as high as 25% [31, 32]. The frequency of LV dysfunction in septic patients can reach 40%, which might result in the increase in PASP via elevated left atrial pressure [2, 3].

Several prior studies reported the association of RV function and the prognosis of ICU patients [4, 5, 26, 33]. However, the prognostic value of the TAPSE/PASP ratio for sepsis and septic shock patients has not been reported. The present study showed that the correlation between TAPSE and PASP was rather low in septic shock patients. This is consistent with a prior study that found that TAPSE and maximal tricuspid regurgitation pressure gradient were not related in a group of heart failure patients [34]. Since both TAPSE and PASP can be affected in septic patients, the TAPSE/PASP ratio has the potential to result in a cumulative risk prediction. Therefore, the TAPSE/PASP ratio deserves more attention in the management of septic shock patients.

We found that the TAPSE/PASP ratio was lower in patients with low LVEF or MAPSE than in patients with high LVEF or MAPSE. Nevertheless, the TAPSE/PASP ratio did not discriminate the three LVEF and MAPSE tertiles as well as it did with the TAPSE tertile. This study also demonstrated that LV systolic function was not associated with prognosis among these patients, which is in line with previous studies [35, 36]. Given that septic cardiomyopathy was diagnosed by LV systolic function, this study indicates that RV function should be taken into consideration in the diagnosis and management of septic cardiomyopathy. Some researchers have reported that left ventricular-arterial uncoupling is common in septic shock patients and has been deemed as a parameter of left ventricular performance [37, 38]. Although few studies have been performed on the association of left ventricular-arterial coupling and the long-term prognosis of septic patients, we speculate that right ventricular-pulmonary arterial coupling may be more clinically relevant in these patients. Future studies are warranted to elucidate this speculation.

### Limitations

This study has several limitations. First, this study was conducted at a single centre, and the sample size was limited. Second, although we incorporated mitral e’ velocity, the LV diastolic function was not fully evaluated. Third, given the nature of this one-time echocardiographic examination, we cannot rule out pre-existing RV dysfunction that could have affected the prognosis of these septic patients. Fourth, the exclusion of patients without TR measurements would cause selection bias. However, the PASP measurement from TR velocity was feasible in most cases. Prior studies have also reported the high obtainment rate of TR measurements [14, 29]. In addition, instead of estimating the CVP from the inferior vena cava diameter, we were able to measure the CVP from a central venous catheter, which would increase the accuracy of PASP estimation.

## Conclusion

The TAPSE/PASP ratio demonstrated prognostic value for one-year all-cause mortality, ICU mortality and MV duration in mechanically ventilated septic shock patients.

## Supplementary information

**Additional file 1: Supplemental Table 1.** Factors associated with ICU mortality. **Supplemental Table 2.** Significant independent relation of MV duration with other variables. **Supplemental Table 3.** Significant independent relation of ICU length of stay with other variables.

## Data Availability

All datasets used and analyzed during the current study are available from the corresponding author on reasonable request.

## Abbreviations

ICU: intensive care unit
MV: mechanical ventilation
TAPSE: tricuspid annular plane systolic excursion
TR: tricuspid regurgitation
PASP: pulmonary arterial systolic pressure
RV: right ventricle
RVOT-FS: right ventricular outflow tract fractional shortening
LVEF: left ventricular ejection fraction
MAPSE: mitral annular plane systolic excursion
APACHE: acute physiology and chronic health evaluation
SOFA: sequential organ failure assessment
HR: heart rate
MAP: mean arterial pressure
CVP: central venous pressure
ICC: intraclass correlation coefficient
PEEP: positive end-expiratory pressure
Pplat: plateau pressure
NE: norepinephrine
