# Supplementary material for: Prognostic implications of tricuspid annular plane systolic excursion/pulmonary arterial systolic pressure ratio in septic shock patients
Source: Cardiovasc Ultrasound. 2020 Jun 12;18:20. doi: 10.1186/s12947-020-00198-y (PMC7293130; doi:10.1186/s12947-020-00198-y)
Supplement: Supplementary file 1 — Additional file 1: Supplemental Table 1. Factors associated with ICU mortality. Supplemental Table 2. Significant independent relation of MV duration with other variables. Supplemental Table 3. Significant independent relation of ICU length of stay with other variables. [file 12947_2020_198_MOESM1_ESM.docx]

Supplemental table 1. Factors associated with ICU mortality

|  | Hazard Ratio | 95%CI | *p* Value |
| --- | --- | --- | --- |
| Univariate analysis |  |  |  |
| Age | 1.002 | 0.974-1.032 | 0.872 |
| SOFA | 1.390 | 1.178-1.641 | <0.001 |
| APACHEII | 1.054 | 1.009-1.102 | 0.019 |
| NE dose | 1.939 | 1.259-2.985 | 0.003 |
| Pplat | 1.076 | 0.984-1.178 | 0.109 |
| TAPSE | 0.361 | 0.157-0.826 | 0.016 |
| PASP | 1.035 | 1.001-1.070 | 0.045 |
| TAPSE/PASP | 0.016 | 0.001-0.213 | 0.002 |
| RVOT-FS | 0.998 | 0.966-1.031 | 0.916 |
| LVEF | 1.002 | 0.9724-1.033 | 0.892 |
| MAPSE | 0.346 | 0.085-1.405 | 0.138 |
| e’ | 0.853 | 0.718-1.014 | 0.071 |
| Multivariable analysis 1 |  |  |  |
| SOFA | 1.365 | 1.096-1.700 | 0.005 |
| Multivariable analysis 2 |  |  |  |
| SOFA | 1.372 | 1.128-1.669 | 0.002 |
| TAPSE/PASP | 0.027 | 0.001-0.530 | 0.017 |

APACHE: acute physiology and chronic health evaluation; SOFA: sequential organ failure assessment; NE: norepinephrine; Pplat: plateau pressure; TAPSE: tricuspid annular plane systolic excursion; PASP: pulmonary arterial systolic pressure; RVOT-FS: right ventricular outflow tract fractional shortening; LVEF: left ventricular ejection fraction; MAPSE: mitral annular plane systolic excursion; e’: mitral e’ velocity.

Supplemental table 2. Significant independent relation of MV duration with other variables

| Variables | | Univariable analysis | | | | |  | | | Multivariable analysis | | |
| --- | --- | --- | --- | --- | --- | --- | --- | --- | --- | --- | --- | --- |
|  |  | r | | 95%CI | *p* | | |  | | Coefficient (β) | | *p* |
| Age | 0.175 | | −0.006-0.344 | | | 0.058 | | |  |  |  | |
| APACHEII | 0.144 | | −0.037-0.317 | | | 0.120 | | |  |  |  | |
| SOFA | 0.082 | | −0.010-0.260 | | | 0.378 | | |  |  |  | |
| TAPSE/PASP | −0.267 | | −0.426-−0.089 | | | 0.004 | | |  | −0.240 | 0.010 | |
| RVOT-FS | −0.270 | | −0.429-−0.092 | | | 0.003 | | |  | −0.311 | 0.001 | |
| LVEF | −0.183 | | −0.353-0.001 | | | 0.049 | | |  |  |  | |
| MAPSE | −0.176 | | −0.350-0.009 | | | 0.060 | | |  |  |  | |
| e’ | −0.202 | | −0.373-−0.017 | | | 0.033 | | |  |  |  | |

APACHE: acute physiology and chronic health evaluation; SOFA: sequential organ failure assessment; TAPSE: tricuspid annular plane systolic excursion; PASP: pulmonary arterial systolic pressure; RVOT-FS: right ventricular outflow tract fractional shortening; LVEF: left ventricular ejection fraction; MAPSE: mitral annular plane systolic excursion; e’: mitral e’ velocity.

Supplemental table 3. Significant independent relation of ICU length of stay with other variables

| Variables | | Univariable analysis | | | | |  | | Multivariable analysis | | |
| --- | --- | --- | --- | --- | --- | --- | --- | --- | --- | --- | --- |
|  |  | r | | 95%CI | *p* | |  |  | Coefficient (β) | | *p* |
| Age | 0.166 | | −0.209 - 0.337 | | | 0.072 | |  | | 0.193 | 0.034 |
| APACHEII | 0.106 | | −0.076 - 0.282 | | | 0.252 | |  | |  |  |
| SOFA | 0.101 | | −0.081 - 0.277 | | | 0.275 | |  | |  |  |
| TAPSE/PASP | −0.206 | | −0.373 - −0.026 | | | 0.025 | |  | |  |  |
| RVOT-FS | −0.210 | | −0.376 - −0.030 | | | 0.022 | |  | | −0.235 | 0.010 |
| LVEF | −0.187 | | −0.358 - −0.005 | | | 0.044 | |  | |  |  |
| MAPSE | −0.118 | | −0.296 - 0.068 | | | 0.213 | |  | |  |  |
| e’ | −0.139 | | −0.316 - 0.048 | | | 0.143 | |  | |  |  |

APACHE: acute physiology and chronic health evaluation; SOFA: sequential organ failure assessment; TAPSE: tricuspid annular plane systolic excursion; PASP: pulmonary arterial systolic pressure; RVOT-FS: right ventricular outflow tract fractional shortening; LVEF: left ventricular ejection fraction; MAPSE: mitral annular plane systolic excursion; e’: mitral e’ velocity.
